# Alteration and the Function of Intestinal Microbiota in High-Fat-Diet- or Genetics-Induced Lipid Accumulation

**DOI:** 10.3389/fmicb.2021.741616

**Published:** 2021-09-17

**Authors:** Fang Qiao, Fang Tan, Ling-Yu Li, Hong-Bo Lv, Liqiao Chen, Zhen-Yu Du, Mei-Ling Zhang

**Affiliations:** Laboratory of Aquaculture Nutrition and Environmental Health (LANEH), School of Life Sciences, East China Normal University, Shanghai, China

**Keywords:** gut microbiota, lipid accumulation, diet, host genetics, 16S rRNA sequencing, obesity

## Abstract

Diet and host genetics influence the composition of intestinal microbiota, yet few studies have compared the function of intestinal microbiota in the diet- or genotype-induced lipid deposition, which limits our understanding of the role of intestinal bacteria in metabolic disorders. The lipid accumulation in wild-type zebrafish fed with control (CON) or high-fat (HF) diet and two gene-knockout zebrafish lines (*cpt1b*^–/–^ or *pparab*^–/–^) fed with control diet was measured after a 4-week feeding experiment. The intestinal microbiota composition of these groups was investigated using 16S ribosomal RNA (rRNA) gene sequencing (DNA-based) and 16S rRNA sequencing (RNA-based). The HF diet or deficiency of two genes induced more weight gain and higher triglyceride content in the liver compared with their control group. 16S rRNA gene sequencing (DNA-based) indicated the decreased abundance of *Proteobacteria* in the HF group compared with CON, but there was no significant difference in bacterial α diversity among treatments. 16S rRNA sequencing (RNA-based) confirmed the decreased abundance of *Proteobacteria* and the bacterial α diversity in the HF group compared with CON. Deficiency of *cpt1b* or *pparab* showed less change in microbiota composition compared with their wild-type group. Intestinal microbiota of each group was transferred to germ-free zebrafish, and the quantification of Nile red staining indicated that the intestinal microbiota of the HF group induced more lipid accumulation compared with CON, whereas intestinal microbiota of *cpt1b*^–/–^ and *pparab*^–/–^ zebrafish did not. The results showed that RNA-based bacterial sequencing revealed more bacterial alteration than DNA-based bacterial sequencing. HF diet had a more dominant role in shaping gut microbiota composition to induce lipid accumulation compared with the gene-knockout of *cpt1b* or *pparab* in zebrafish, and the transplant of intestinal microbiota from HF-fed fish induced more lipid deposition in germ-free zebrafish. Together, these data suggested that a high-fat diet exerted a more dominant role over the deletion of *cpt1b* or *pparab* on the intestinal bacterial composition, which corresponded to lipid accumulation.

## Introduction

Obesity is one of the most prevalent global challenges, as it increases the risk of a wide range of chronic diseases (Brial et al., 2018; Indiani et al., 2018; Moen et al., 2018). It is well-known that both diet components and genetic factors play important roles in determining an individual’s predisposition to weight gain and being obese (Goodarzi, 2018). Increasing pieces of evidence also suggested that intestinal microbiota was indispensable for the development of obesity. The conventionalization of germ-free mice with the microbiota from obese mice caused substantially higher adiposity than those conventionalized with the microbiota from lean ones (Bäckhed et al., 2004; Turnbaugh et al., 2008). Several bacteria have been found to contribute to lipid accumulation by different mechanisms in mammals. For example, *Lactobacillus paracasei* produces L-lactate, which is converted by intestinal epithelial cells into fatty acids and, subsequently, increases lipid storage in intestinal epithelial cells, whereas *Escherichia coli* produces acetate, which activates genes involved in fatty acids oxidation (Araujo et al., 2020).

Being the substrates for the host and microbiota, diet components influence the intestinal microbial composition and lipid deposition (Landgraf et al., 2017; Araujo et al., 2020). Except for affording more energy, it was found that a high-fat diet diminished gut barrier-protecting bacteria and enriched endotoxin producers (Chassaing et al., 2015) to induce lipid accumulation (Faillaci et al., 2018). Although the intestinal microbiota is influenced by diet components, there is a substantial interindividual variation in the intestinal bacterial composition in response to diets, suggesting the genetic factor should not be ignored when we evaluate the metabolic characteristics (Ussar et al., 2016; Singh et al., 2019). Kreznar et al. (2017) characterized the metabolic phenotypes of eight genetically distinct inbred mouse strains in response to a high energy diet and found that different mouse strains showed great variation in the intestinal microbiota as well as the diabetes-related phenotypes, showing the dominant role of genetic factors in shaping the intestinal microbiota. Gut microbiota, which acts as a “second genome” for modulating the nutrition, metabolism, and immunity phenotypes of the superorganism host (Jia et al., 2008), is likely indispensable for obesity development. It has been shown that the presence of diet enriched the bacteria from the phylum Firmicutes, which were sufficient to increase the number of intestinal epithelial lipid droplets in zebrafish (Semova et al., 2012). However, few studies have compared the function of intestinal microbiota in diet-induced or genotype-induced lipid deposition.

As in mammals, fish also accumulate extra lipid when fed with a high-fat diet (Zhang et al., 2020) and the knockout of genes related to lipid catabolism also cause lipid accumulation in zebrafish (Li et al., 2020). Identifying the contribution of intestinal microbiota in diet- or genetic-induced obesity in fish, which are more primitive than mammals, may afford more evolutionary information on the complex relationship among nutrients intake, genetic factors, intestinal microbiota, and the development of obesity.

16S ribosomal RNA (rRNA) gene amplicon sequencing is widely used to reflect the bacterial composition, but it should be noticed that a high abundance of certain bacteria not necessarily implies the high activity of these bacteria (De Vrieze et al., 2018). 16S rRNA sequencing is also used to estimate the active bacterial members, and plenty of studies indicate the existence of a difference between the DNA and the RNA profiles, suggesting alternative techniques including intestinal microbiota transplant or metabolomics should be involved to identify the function of microbiota (Franchi et al., 2018).

In the present study, the relative contributions of intestinal microbiota in genotype- or diet-induced lipid accumulation were detected in zebrafish. Two gene-knockout models (*cpt1b*^–/–^ and *pparab*^–/–^) were generated. Carnitine acyltransferase 1b (*cpt1b*) is a limiting enzyme of fatty acid β-oxidation, which plays a crucial role in transferring long-chain fatty acid from CoA to carnitine for oxidation in the mitochondrion (Li et al., 2018). Peroxisome proliferator-activated receptor α is one of the nuclear receptors that promotes fatty acid β-oxidation and regulates lipid homeostasis (Madureira et al., 2017). Wild-type (WT) zebrafish were fed with different energy diets (control diet or high-fat diet) and different genotypes (*cpt1b*^–/–^ and *pparab*^–/–^). Zebrafish were fed with the control diet for 4 weeks. The lipid accumulation phenotypes were measured, and the intestinal microbiota composition was analyzed based on 16S rRNA gene (DNA-based) and 16S rRNA (RNA-based) sequencing. To detect the bacterial function, the intestinal microbiota of adult zebrafish was collected from each experimental group (CON vs. HF, WT vs. *cpt1b*^–/–^, WT vs. *pparab*^–/–^) and transplanted to larval germ-free zebrafish to identify whether intestinal microbiota reshaped by different diet components or genetic background could cause lipid accumulation in germ-free zebrafish.

## Materials and Methods

### Ethics Statement and Consent to Participate

All experiments were performed under the Guidance for the Care and Use of Laboratory Animals in China. Laboratory Animals in China. This study was approved by the Committee on the Ethics of Animal Experiments of East China Normal University (No. F20190101).

### Animal Husbandry

The WT adult AB line zebrafish (4–5 months) used in the experiment were obtained from the Chinese National Zebrafish Resource Center (Wuhan, China). Zebrafish were reared in a zebrafish breeding recirculating system under a 14-h light–10-h dark cycle at 28°C. Dissolved oxygen and nitrogen were 6.07 ± 0.01 and 0.11 ± 0.01 mg/L, respectively. The fish were fed twice daily (09:00 and 17:00) using a commercial diet (Shengsuo, Yantai, China) containing 50% protein and 7% lipid. One week before the experiment, the male fish were selected for the treatments to avoid the sex differences in metabolism.

### Establishment of *cpt1b* and *pparab* Mutant Zebrafish Lines

The *cpt1b*^–/–^ and *pparab*^–/–^ zebrafish were generated using CRISPR/Cas9 gene-editing technology as described in our previous studies (Lu D. L. et al., 2019; Li et al., 2020).

### Feeding Experiments and Sampling

The WT zebrafish were randomly divided into two groups. They were fed with a CON diet (7% fat) and HF diet (15% fat) (Table 1). The *cpt1b*^–/–^, *pparab*^–/–^ zebrafish and their matrilineal WT fish were fed with the control diet. Each treatment had three replicate tanks (40 fish per tank). Fish were fed twice daily at 9:00 am and 5:00 pm at a ratio of 4% of the fish weight of each tank for 4 weeks. Fish were weighed every week to plot the weight gain curve. At the end of the feeding trial, all fish were fasted for 24 h and then sampled. Fifteen fish of each treatment were randomly collected to measure the body length and body weight to determine the condition factor (CF; CF = body weight/body length^3^) and then used for the total lipid extraction. Twelve fish of each treatment were dissected on ice to obtain the liver and muscle for the subsequent biochemical analysis.

**TABLE 1:** The formulation and proximate composition of the experimental diet.

Dietary ingredient (g kg^–1^)	CON	HF
Casein	320	320
Gelatin	80	80
Corn starch	300	300
Soybean oil	70	150
Vitamin premix^a^	10	10
Mineral premix^b^	30	30
Choline chloride	5	5
Butylated hydroxytoluene	0.25	0.25
Dimethyl-beta-propiothetin	2	2
Ca(H_2_PO_4_)_2_	15	15
Carboxymethyl cellulose	30	30
Cellulose	137.75	57.75
Proximate composition		
Moisture (%)	9.09	9.59
Total fat (% dry matter)	6.68	14.23
Total protein (% dry matter)	38.25	37.93
Ash (% dry matter)	2.98	2.67

*^a^Vitamin premix (mg or IU/kg): 7,000,000 I.U. (international units) Vitamin A, 1,500,000 I.U. Vitamin D3, 15,000 mg Vitamin E, 6,000 mg Vitamin K3, 8,000 mg Vitamin B1, 10,000 mg Vitamin B2, 10,000 mg Vitamin B6, 20 mg Vitamin B12, 60,000 mg Inositol, 20,000 mg calcium pantothenate, 35,000 mg niacinamide, 1,000 mg Folic acid, 100 mg Biotin, 50,000 mg Vitamin C, and bran powder, etc.*

*^b^Mineral premix (g or mg/kg): 40 g Fe, 12 g Zn, 3.6 g Cu, 4 g Mn, 20 g Mg, 25 g K, 400 mg Co, 300 mg I, and zeolite powder, etc. Mineral premix and Vitamin premix were bought from Zhejiang Minsheng Biotechnology Co., Ltd.*

### Determination of the Contents of Total Lipid and Triglyceride

Total lipid content was measured using the classic methanol–chloroform method (Bligh and Dyer, 1959). The triglyceride (TG) contents of the liver and muscle were determined with commercial kits according to the manufacturer’s instructions (Nanjing Jiancheng Bioengineering Institute, Nanjing, China). Protein concentration was determined with the Enhanced BCA Protein Assay Kit (Beyotime, Biotechnology, Shanghai, China).

### Bacterial DNA and RNA Extraction and Amplification

Bacterial DNA was extracted from approximately 0.1 g of intestinal contents of a mixture of five fish using QlAamp DNA Stool Mini Kit 51604 (QIAGEN, Hilden, Germany) following the manufacturer’s instructions. RNA was extracted from approximately 0.1 g of intestinal content of a mixture of five fish, according to a previously described method (Zhang Z. et al., 2019) with some modifications. Co-extracted DNA was removed using RNase-free DNase (QIAGEN, Hilden, Germany) according to the manufacturer’s instructions. The resulting RNA was purified using an RNeasy^®^ Mini Kit (QIAGEN, Hilden, Germany). Purified RNA of intestinal microbiota was used for complementary DNA synthesis using a PrimeScript^TM^ RT-PCR Kit (Takara, Dalian, China) according to the instruction of the manufacturers. The final quality of the complementary DNA and DNA was checked by 1% agarose gel electrophoresis. The amplicons were subsequently purified and analyzed on an Illumina Miseq Platform (Personal Biotechnology Co., Ltd., Shanghai, China) by sequencing the V3-V4 16S rRNA region, using the primers 338F 5-ACTCCTACGGGAGGCAGCA-3 and 806R 5-GGACTACHVGGGTWTCTAAT-3. To distinguish DNA and RNA samples, each reverse primer was tagged with a unique 6-bp unique barcode. The amplification reaction and thermal cycles were described in a previous study (Lu H. F. et al., 2019). Microbiota sequencing data were submitted to National Center for Biotechnology Information with the accession number PRJNA632454.

### High-Throughput Sequencing and Analyses

A nonparametric Mann–Whitney *U* test was used to test the significant differences among groups. Principal component analyses based on unweighted UniFrac distance metrics were conducted, and the R package was used to visualize interactions among the bacterial communities from different samples. Bacterial richness and diversity across the samples were calculated using Chao1, ACE, Shannon, and Simpson richness estimators (Oksanen et al., 2014; Lu H. F. et al., 2019). Spearman’s correlation analyses were used to assess the potential association between the bacterial genera and the lipid deposition phenotype in zebrafish using Hmisc package in R (McMurdie and Holmes, 2013).

### Production of Germ-Free Zebrafish and Gut Microbiota Transplantation

The production of germ-free zebrafish was performed following established protocols as previously described (Pham et al., 2008; Tan et al., 2019). To determine the function of gut microbiota in lipid accumulation, the intestinal bacterial cells collected from zebrafish in different treatments were added to the gnotobiotic zebrafish medium containing 3 days post-fertilization (dpf) germ-free zebrafish at a concentration of 10^4^ CFUs/ml (Tan et al., 2019) and the inoculation lasted for 7 days. The zebrafish were fasted for 12 h before Nile Red staining.

### Nile Red Staining

Nile Red powder (Sangon Biotech, Shanghai, China) was dissolved in acetone at a concentration of 1 mg/ml as the stock solution. Larvae were immersed in system water containing 0.1 mg/ml Nile Red stock for 1 h at room temperature in the dark. Afterward, the stained larvae were washed with fresh medium three times to remove Nile red adhering to the surface. Images were obtained using an ECLIPSE Ti2 fluorescence microscope (Nikon, Tokyo, Japan) at an excitation wavelength of 543 nm. The images were converted to an 8-bit grayscale for measuring mean gray value using ImageJ software (National Institutes of Health). The fluorescent area and intensity of images were quantified using ImageJ to evaluate the lipid accumulation degree of larvae.

### Statistical Analyses

All data were presented as mean ± SEM. Student’s unpaired *t*-test analyzed the significant difference. All statistical analyzes were performed using the Statistical Package for the Social Sciences version 20.0 software for Windows (IBM, Armonk, NY, United States). Results with a *p*-value < 0.05 were considered statistically significant. Correlations with *p-*values lower than 0.05 were considered significant.

## Results

### Both High Energy Diets and Deficiency of Lipid Catabolism Related Genes Induced More Lipid Accumulation in Zebrafish

**FIGURE 1:**
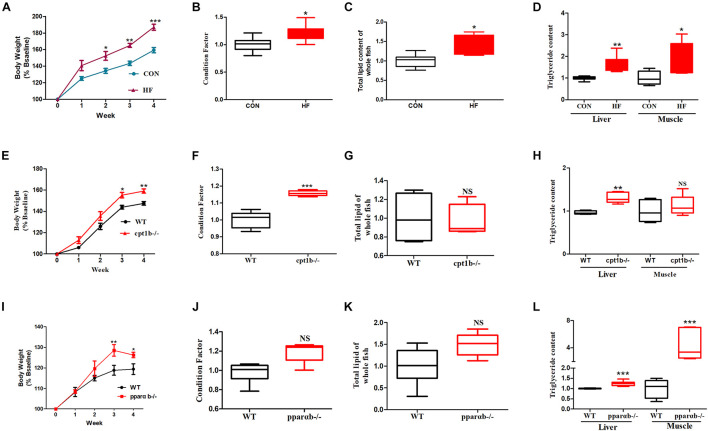
Phenotypes of zebrafish with different genotypes or fed on different diets. **(A)** Growth curve established using average bodyweight of two dietary groups (CON and HF) every week for 4 weeks (*n* = 3 per group). **(B)** Condition factor (CF) of two dietary groups (*n* = 15 per group). **(C)** Total lipid of whole fish of two dietary groups (*n* = 15 per group). **(D)** Triglyceride contents of liver and muscle of two groups (*n* = 8 per group). **(E)** Growth curve established using average bodyweight of WT and *cpt1b*^–/–^ zebrafish every week for 4 weeks (*n* = 3 per group). **(F)** Condition factor of WT and *cpt1b*^–/–^ zebrafish (*n* = 15 per group). **(G)** Total lipid of whole fish of WT and *cpt1b*^–/–^ zebrafish (*n* = 15 per group). **(H)** Triglyceride contents in liver and muscle of WT and *cpt1b*^–/–^ zebrafish (*n* = 8 per group). **(I)** Growth curve established using average bodyweight of WT and *pparab*^–/–^ zebrafish every week for 4 weeks (*n* = 3 per group). **(J)** Condition factor of WT and *pparab*^–/–^ zebrafish (*n* = 15 per group). **(K)** Total lipid of whole fish of WT and *pparab*^–/–^ zebrafish (*n* = 15 per group). **(L)** Triglyceride contents in liver and muscle of WT and *pparab*^–/–^ zebrafish (*n* = 8 per group). All data are normalized to control group (100%) and presented as means ± SEM. Significance difference was analyzed by Student’s *t*-test (*p < 0.05, **p < 0.01, ***p < 0.001).

During the feeding trial, WT AB line zebrafish fed with the HF diet gained more weight and showed significantly increased values of CF, which was supposed to be higher in fatter fish (Landgraf et al., 2017), compared with those fed with the CON diet (Figures 1A,B). Furthermore, the HF group had higher contents of total lipid of whole fish and TG in the liver and muscle compared with the CON group (Figures 1C,D), suggesting that a high-fat diet induced extra lipid accumulation in zebrafish. After ingesting the control diet for 4 weeks, *cpt1b*^–/–^ zebrafish gained more bodyweight (Figure 1E) and showed increased values of CF relative to WT zebrafish (Figure 1F). Total lipid content of whole fish showed no significant difference between WT and *cpt1b^–/–^* zebrafish (Figure 1G), whereas *cpt1b*^–/–^ zebrafish presented higher TG level in the liver (Figure 1H). The growth curve revealed that *pparab*^–/–^ zebrafish gained higher body weight than WT zebrafish (Figure 1I). Both the values of CF and the contents of total lipid were higher in *pparab*^–/–^ zebrafish, but no significant difference was found (Figures 1J,K). Furthermore, *pparab*^–/–^ zebrafish showed higher TG contents in the liver and muscle (Figure 1L). These data indicated that compared with WT zebrafish, *cpt1b*^–/–^ and *pparab*^–/–^ zebrafish were characterized by a significantly higher degree of lipid accumulation.

### Influence of Diets and Gene Manipulation on Gut Microbial Diversity Based on 16S Ribosomal RNA Gene and 16S Ribosomal RNA Sequencing

**FIGURE 2:**
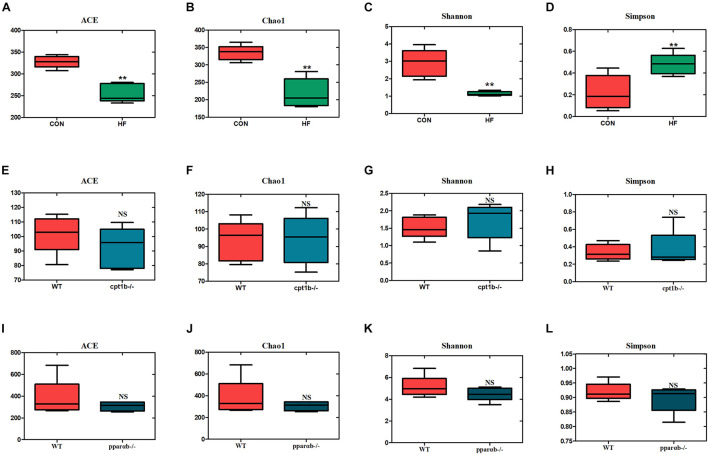
Boxplots of α diversity indices of bacterial community based on 16S rRNA sequencing. **(A–D)** Gut microbial α diversity of dietary groups based on **(A)** ACE, **(B)** Chao1, **(C)** Shannon, and **(D)** Simpson. **(E–H)** Gut microbial α diversity of WT and *cpt1b*^–/–^ zebrafish groups based on **(E)** ACE, **(F)** Chao1, **(G)** Shannon, and **(H)** Simpson. **(I–L)** Gut microbial α diversity of WT and *pparab*^–/–^ zebrafish based on **(I)** ACE, **(J)** Chao1, **(K)** Shannon, and **(L)** Simpson. All data are normalized to control group (100%) (**p < 0.01).

To investigate the responses of intestinal microbiota to different diets or gene mutants, the microbial composition of each experimental group was analyzed by high-throughput sequencing based on microbial 16S rRNA gene and 16S rRNA. In total, samples based on total microbial analysis yielded between 60,423 to 75,919 reads per sample with an average of 66,031 sequences per sample [2,200 operational taxonomic units (OTUs) per sample]. For samples of 16S rRNA analysis, we obtained 61,599 to 74,862 OTUs per sample in total, with an average of 70,282 reads per sample (around 1,400 OTUs per sample). DNA-based analyses indicated no significant difference in α diversity between the dietary challenge groups or manipulating genetic groups according to ACE, Chao1, Shannon, and Simpson richness estimators (Supplementary Figure 1). RNA-based microbial data showed a less diverse microbial community in the HF group relative to the CON group (*p* < 0.001) (Figures 2A–C). The HF group showed a significantly higher level of diversity based on the Simpson richness indices (Figure 2D). In contrast, no significant difference was found among these diversity indexes when WT was compared with the *cpt1b*^–/–^ group (*p* > 0.05) (Figures 2E–H) or WT relative to the *pparab*^–/–^ group (*p* > 0.05) (Figures 2I–L). In summary, the active microbial community captured the decline of gut microbial α diversity in the HF group compared with the CON group, whereas DNA-based microbial sequence reflected no evident difference in α diversity among all experimental groups (Supplementary Figure 1).

### Effects of Diets and Gene Manipulation on Gut Microbial Composition

**FIGURE 3:**
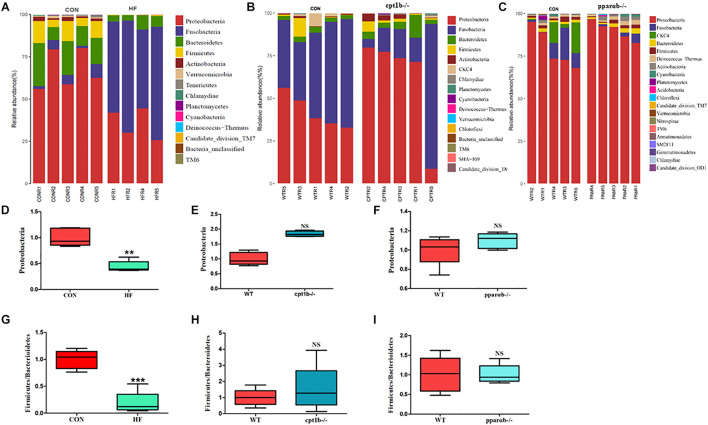
Relative phyla abundance of microbiome based on 16S rRNA gene sequencing. **(A)** Relative abundances of phylum level among dietary groups (CON and HF). **(B)** Relative abundances of phylum level between WT and *cpt1b*^–/–^ zebrafish. **(C)** Relative abundances of phylum level between WT and *pparab*^–/–^ zebrafish. **(D–F)** Ratio of *Firmicutes*/*Bacteroidetes* in gut microbiota of **(D)** dietary groups, **(E)** WT and *cpt1b*^–/–^ zebrafish, and **(F)** WT and *pparab*^–/–^ zebrafish. **(G–I)** Ratio of *Fusobacteria*/*Proteobacteria* in gut microbiota of **(G)** dietary groups, **(H)** WT and *cpt1b*^–/–^ zebrafish, and **(I)** WT and *pparab*^–/–^ zebrafish. All data are normalized to control group (100%) and presented as means ± SEM (**p < 0.01, ***p < 0.001).

According to DNA-based analysis, all identified reads from the intestinal samples belonged to 20 phyla, and over 70% of sequences belonged to two phyla (*Proteobacteria* and *Fusobacteria*), followed by *Bacteroidetes* and *Firmicutes* (Figures 3A–C). Intriguingly, the abundance of *Proteobacteria* in the HF group remarkably decreased compared with the CON group (Figure 3D). The abundance of *Proteobacteria* showed no difference between genes manipulated groups (*cpt1b*^–/–^ or *pparab*^–/–^) and the WT group (Figures 3E,F). The effects of host genetics and dietary components on the ratio of *Bacteroidetes* to *Firmicutes* were also estimated, and no significant difference was found (Figures 3G–I).

**FIGURE 4:**
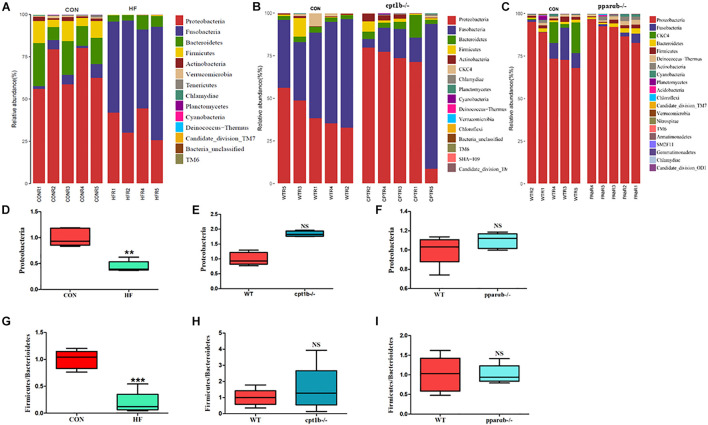
Relative phyla abundances of microbiome based on 16S rRNA sequencing. **(A)** Relative abundances of phylum level of two dietary groups (CON and HF). **(B)** Relative abundances of phylum level between WT and *cpt1b*^–/–^ zebrafish. **(C)** Relative abundances of phylum level between WT and *pparab*^–/–^ zebrafish. **(D–F)** Ratio of *Firmicutes*/*Bacteroidetes* in gut microbiota of **(D)** dietary groups, **(E)** WT and *cpt1b*^–/–^ zebrafish, and **(F)** WT and *pparab*^–/–^ zebrafish. **(G–I)** Ratio of *Fusobacteria*/*Proteobacteria* in gut microbiota of **(G)** dietary groups, **(H)** WT and *cpt1b*^–/–^ zebrafish, and **(I)** WT and *pparab*^–/–^ zebrafish. All data are normalized to control group (100%) and presented as means ± SEM (**p < 0.01, ***p < 0.001).

At the RNA level, 17 bacterial phyla were found in the zebrafish gut, and most of the identified reads belong to two phyla, *Proteobacteria* and *Fusobacteria*, followed by *Bacteroidetes* and *Firmicutes*, which was similar to DNA-based sequencing analysis (Figures 4A–C). A significant decrease in the abundance of *Proteobacteria* was also observed in the HF group compared with the CON group, whereas there was no significant distinction of that between genes manipulated groups (*cpt1b*^–/–^ or *pparab*^–/–^) and the WT group (Figures 4D–F). Inconsistent with the total microbial analysis, the active microbial analysis illustrated that HF diet significantly reduced the ratio of *Firmicutes* to *Bacteroidetes* vs. CON diet (Figure 4G), but no dramatic difference of that was detected in *cpt1b*^–/–^ or *pparab*^–/–^ compared with the WT group (Figures 4H,I). These results demonstrated that although dietary interruption and gene manipulation all induced extra lipid accumulation in zebrafish, only the HF diet reduced the abundance of *Proteobacteria*.

### Correlation of Intestinal Microbiota Community and Lipid Accumulation

**FIGURE 5:**
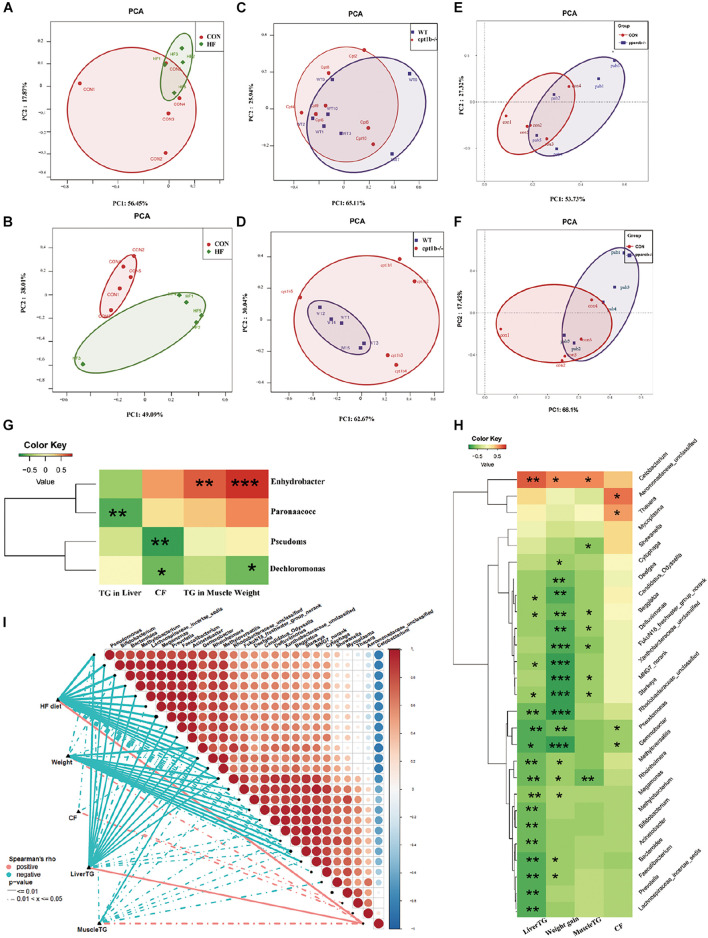
Comparison of gut microbiota composition among different dietary groups and different genotypes on same diets. **(A)** Principal components analysis (PCA) scores plot of dietary groups (CON and HF) based on 16S rRNA genes (*n* = 5 per group). **(B)** PCA scores plot of dietary groups (CON and HF) based on 16S rRNA (*n* = 5 per group). **(C)** PCA scores plot of WT and *cpt1b*^–/–^ zebrafish based on 16S rRNA genes (*n* = 5 per group). **(D)** PCA scores plot of WT and *cpt1b*^–/–^ zebrafish based on 16S rRNA (*n* = 5 per group). **(E)** PCA scores plot of WT and *pparab*^–/–^ zebrafish based on 16S rRNA genes (*n* = 5 per group). **(F)** PCA scores plot of WT and *pparab*^–/–^ zebrafish based on 16S rRNA (*n* = 5 per group). **(G,H)** Heatmap illustrates Spearman’s correlation between microbial genus and lipid overaccumulation traits measured between control and high-fat diet group based on **(G)** total microbial analysis and **(H)** active microbial analysis. **(I)** Correlation between abundance of microbial genus and lipid overaccumulation traits in HF groups based on active microbial analysis. *p < 0.05, **p < 0.01, ***p < 0.001.

The intestinal microbiota in the HF diet group differed from the control group (Figures 5A,B), whereas it showed no separation between genes-manipulated groups and WT based on active or total microbial community (Figures 5C–F). Considering that dietary interruption caused more significant changes of intestinal microbiota, Spearman’s rank correlation was performed to identify the correlation between the gut bacterial genus and lipid overaccumulation parameters between the control and high-fat diet groups. At the DNA level, excessive lipid accumulation or weight gain induced by the HF diet was positively related to *Dechloromonas* (Spearman’s correlation > 0.3, *p* < 0.05) and negatively associated with *Enhydrobacte*, *Paracoccus*, and *Pseudomonas* (Spearman’s correlation < −0.3, *p* < 0.05) (Figure 5G). At the RNA level, three genus-level taxa *Cetobacteria*, *Aeromonadaceae*, and *Thauera* were positively correlated with lipid accumulation or higher weight phenotype, whereas 24 genus-level taxa, including *Bifidobacterium*, *Bacteroides*, and *Prevotella*, were negatively relevant to lipid overaccumulation (Figure 5H), suggesting the active microbial sequencing revealed more bacteria genus associated with the lipid deposition in dietary treatments. *Cetobacteria* and *Pseudomonas* were the most abundant among 27 microbial genera, and the abundance of *Cetobacteria* was negatively linked with *Pseudomonas* (Figure 5I).

### Intestinal Microbiota Reshaped by a High-Fat Diet Led to Lipid Accumulation in Germ-Free Zebrafish

**FIGURE 6:**
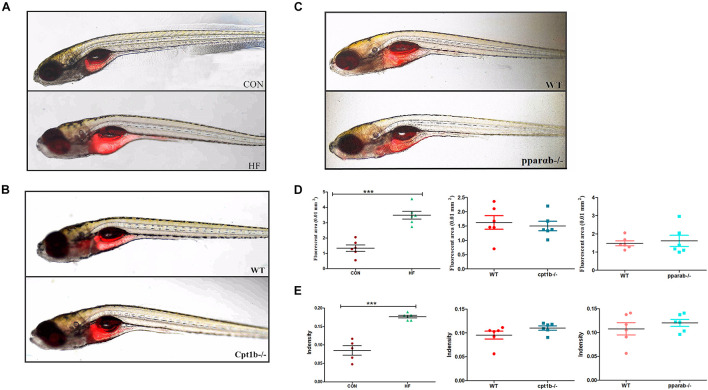
Nile red staining visualizes deep tissue fat deposition in zebrafish. **(A–C)** Fluorescent images of adipose tissue using Nile red staining of **(A)** CON and HF zebrafish, **(B)** WT and *cpt1b*^–/–^ zebrafish, and **(C)** WT and *pparab*^–/–^ zebrafish. **(D,E)** Quantitative evaluation of fluorescent area **(D)** and intensity **(E)** of Nile red staining (*n* = 6 per group). All data are presented as means ± SEM (***p < 0.001).

To further identify the causal relationship of the microbiota in the lipid accumulation, we colonized germ-free zebrafish at 3 dpf with the intestinal microbiota of adult zebrafish collected from each experimental group (CON vs. HF, WT vs. *cpt1b*^–/–^, WT vs. *pparab*^–/–^). The lipid accumulation phenotype of zebrafish larvae was detected by Nile Red staining. The statistical analyses of fluorescent area and intensity showed that the microbiome of the HF group induced a significantly higher degree of lipid accumulation in germ-free zebrafish than that of the CON group (Figures 6A,D,E). The colonization of microbiota with the intestinal microbiota from *cpt1b*^–/–^ zebrafish or *pparab*^–/–^ zebrafish caused no significant difference in lipid accumulation compared with WT zebrafish (Figures 6B–E), implying that intestinal microbes reshaped by HF diet donated more to lipid accumulation in zebrafish larvae compared with microbiota from groups of gene manipulation.

## Discussion

Identifying the interaction among the host genetics, diet, or intestinal microbiota is indispensable for revealing the pathogenesis of obesity. Differences in host genotypes that affect the carbohydrate landscape altered the intestinal bacterial composition in a diet-dependent manner (Kashyap et al., 2013). ob/ob gene mutant caused a major change to the mouse gut microbiota composition than dietary composition (Kashani et al., 2019). Zhang et al. (2010) assessed the relative contributions of host genetics and diets in shaping the intestinal microbiota in mice and indicated that diet could explain 57% of the total structure variation in microbiota, whereas genetic mutation explained for <12%, but the contribution of intestinal microbiota in diet or genotype induced obesity remains unclear. In the present study, we compared the intestinal bacteria in different diet treatments and gene-knockout zebrafish, and we found high-fat diet had a more dominant role in shaping microbiota community to increase the lipid accumulation relative to *cpt1b* and *pparab* knockout, and the follow-up microbiota transplant experiment also confirmed this conclusion. These results suggested that high-fat-diet induced lipid accumulation dependent on the intestinal microbiota, whereas two gene-knockout zebrafish accumulated more lipid mainly by inhibiting lipid catabolism instead of the alteration of intestinal microbiota.

The characteristics of intestinal microbiota in the different types of obesity have been addressed; for example, the decrease of microbial diversity is related to the development of obesity in mammals (Walters et al., 2014). In the present study, active microbial analyses indicated that HF diet had remarkably decreased microbial diversity, but the knockout of *cpt1b* and *pparab* caused no significant difference in microbial diversity, revealing lower bacterial diversity was more prone to exist in a high-fat diet that induced lipid accumulation. Another characteristic related to obesity is the increased ratio of *Firmicutes* to *Bacteroidetes* in mammals (Ley et al., 2006), although different views exist (Schwiertz et al., 2010). In the present study, no increase of *Firmicutes*/*Bacteroidetes* ratio was observed in zebrafish with diet-induced or genotype-induced lipid accumulation based on 16S rRNA gene sequencing. However, 16S rRNA sequencing indicated that HF diet had reduced *Firmicutes*/*Bacteroidetes* ratio, suggesting different sequencing methods also influenced the results. Furthermore, we also observed that the abundance of *Proteobacteria* was significantly declined in the HF group compared with the control group according to both total and active microbial sequencing. *Proteobacteria* is the dominant member in the intestine of numerous aquatic organisms, and it is one of the most responsive groups when the host health is affected by environmental factors such as diet (Navarro-Barron et al., 2019). These results suggested that decreased bacterial diversity and alteration of some responsive bacterial groups to the diet component might be related to diet-induced obesity.

A recent study found nuclear factor-kB/Relish was vital to adapt gut microbiota species composition to host diet macronutrient composition, suggesting the importance of host genetic factors and diet components in shaping microbiota diversity (Vandehoef et al., 2020). The fecal bacteria of Fut2^–^ mice that lack fucosylated host glycans showed decreased alpha diversity relative to Fut2^+^ mice and exhibited significant differences in community composition (Kashyap et al., 2013). The present study showed the intestinal bacteria of *cpt1b*^–/–^ or *pparab*^–/–^ zebrafish exhibit no significant difference relative to WT zebrafish. These results suggested that different genes played different roles in shaping the intestinal microbiota composition and the response of different bacteria to the environmental or genetic factors also varied. More gene manipulation models should be conducted to reveal the influence of host genetics on intestinal microbiota and host metabolism.

The correlation analyses based on DNA level suggested *Enhydrobacter*, *Paracoccus*, *Pseudomonas*, and *Dechloromonas* (*p* < 0.05, Spearman’s rank correlation) were related to lipid accumulation, and among them, *Enhydrobacter* was proved to be able to ferment glucose and carbohydrates in the intestines of shrimp and crabs (Premalatha et al., 2015), but the function of the other three bacterial genera in nutrients metabolism is unknown. The correlation analyses based on 16S rRNA sequencing showed that *Bacteroides* and *Prevotella* negatively related to TG in the liver and weight gain. It was found that *Bacteroides* was negatively correlated with body weight in mice (Sidiropoulos et al., 2020), and the abundance of *Prevotellaceae* was negatively related to a high-fat diet in pigs and humans (Zhang C. et al., 2019; Zhou et al., 2019). Additionally, *Bifidobacterium* and *Faecalibacterium* were negatively related to TG in the liver. These results suggested that the relationship of *Bacteroides*, *Prevotella, Bifidobacterium*, and *Faecalibacterium* to lipid accumulation was conserved among different animals. *Cetobacteria* was positively correlated with lipid accumulation or higher weight phenotype. It has been found that *Cetobacteria* is the producer of acetic acid from peptones or carbohydrates in the fish gut (Qin et al., 2017; Siriyappagouder et al., 2018), which may account for harvesting more energy from the diet.

Amplicon sequencing methods targeting the 16S rRNA gene and 16S rRNA have been extensively applied to investigate the microbial community composition and dynamics in multiple places, including anaerobic digestion (De Vrieze et al., 2018), the rumen of beef cattle (Li F. et al., 2019), a coastal microbial mat (Cardoso et al., 2017), or soils (Bastida et al., 2017; Li H. et al., 2019). Although some differences exist between the total and the active microbial communities, active microbial communities are more sensitive to address community compositions and microbial function predictions (Li F. et al., 2019; Li H. et al., 2019). The present study showed that 16S rRNA-based sequencing result was more consistent with the intestinal bacteria transplantation, suggesting multiple sequencing platform and bacterial transplantation was indispensable for identifying the bacterial function. Bacteria transplantation combined with Nile red staining was used to show the lipid accumulation in zebrafish, but due to the small size of zebrafish (10 dpf), the lack of physiological and biochemical indexes was the limitation of the present study.

## Conclusion

In summary, we found that the intestinal microbiota composition shaped by a high-fat diet played a more important role in lipid accumulation compared with those of *cpt1b^–/–^* and *pparab^–/–^*. 16S rRNA sequencing provided more consistent and accurate information in reflecting the function of the intestinal microbiome relative to 16S rRNA gene sequencing. The interactions among the host genotypes, intestinal microbiota, and diet components should be considered when we identify the development of obesity and its related metabolic syndrome.

## Data Availability Statement

Microbiota sequencing data was submitted to the National Center for Biotechnology Information (NCBI) with the accession number PRJNA632454.

## Ethics Statement

This study was approved by the Committee on the Ethics of Animal Experiments of East China Normal University (No. F20190101).

## Author Contributions

M-LZ and Z-YD conceived the study, designed the experiments, and revised the manuscript. FQ, FT, and L-YL performed the experiments and analyzed the data. L-YL generated the *cpt1b* knockout zebrafish. L-YL and H-BL generated the *pparab* knockout zebrafish. LQC provided the essential materials and technical support. M-LZ and FT wrote the manuscript. All authors contributed experimental assistance, intellectual input to this study, and read and approved the final manuscript.

## Conflict of Interest

The authors declare that the research was conducted in the absence of any commercial or financial relationships that could be construed as a potential conflict of interest.

## Publisher’s Note

All claims expressed in this article are solely those of the authors and do not necessarily represent those of their affiliated organizations, or those of the publisher, the editors and the reviewers. Any product that may be evaluated in this article, or claim that may be made by its manufacturer, is not guaranteed or endorsed by the publisher.
