# Supplementary material for: Alteration and the Function of Intestinal Microbiota in High-Fat-Diet- or Genetics-Induced Lipid Accumulation
Source: Front Microbiol. 2021 Sep 17;12:741616. doi: 10.3389/fmicb.2021.741616 (PMC8484964; doi:10.3389/fmicb.2021.741616)
Supplement: Supplementary file 1 [file Data_Sheet_1.docx]

**The alteration and the function of intestinal microbiota in high-fat-diet- or genetics-induced lipid accumulation**

Fang Qiao#, Fang Tan#,Ling-Yu Li, Hong-Bo Lv, Liqiao Chen, Zhen-Yu Du^*^, Mei-Ling Zhang*,

LANEH,School of Life Sciences, East China Normal University, Shanghai, 200241, China

#Contributed equally to this work.

*Corresponding author:

Prof. Mei-Ling Zhang (Email: mlzhang@bio.ecnu.edu.cn,Tel/Fax: 86-21-54345354)

Prof. Zhen-Yu Du (Email: zydu@bio.ecnu.edu.cn, Tel/Fax: 86-21-54345354)

**Supplementary Figure 1**

**
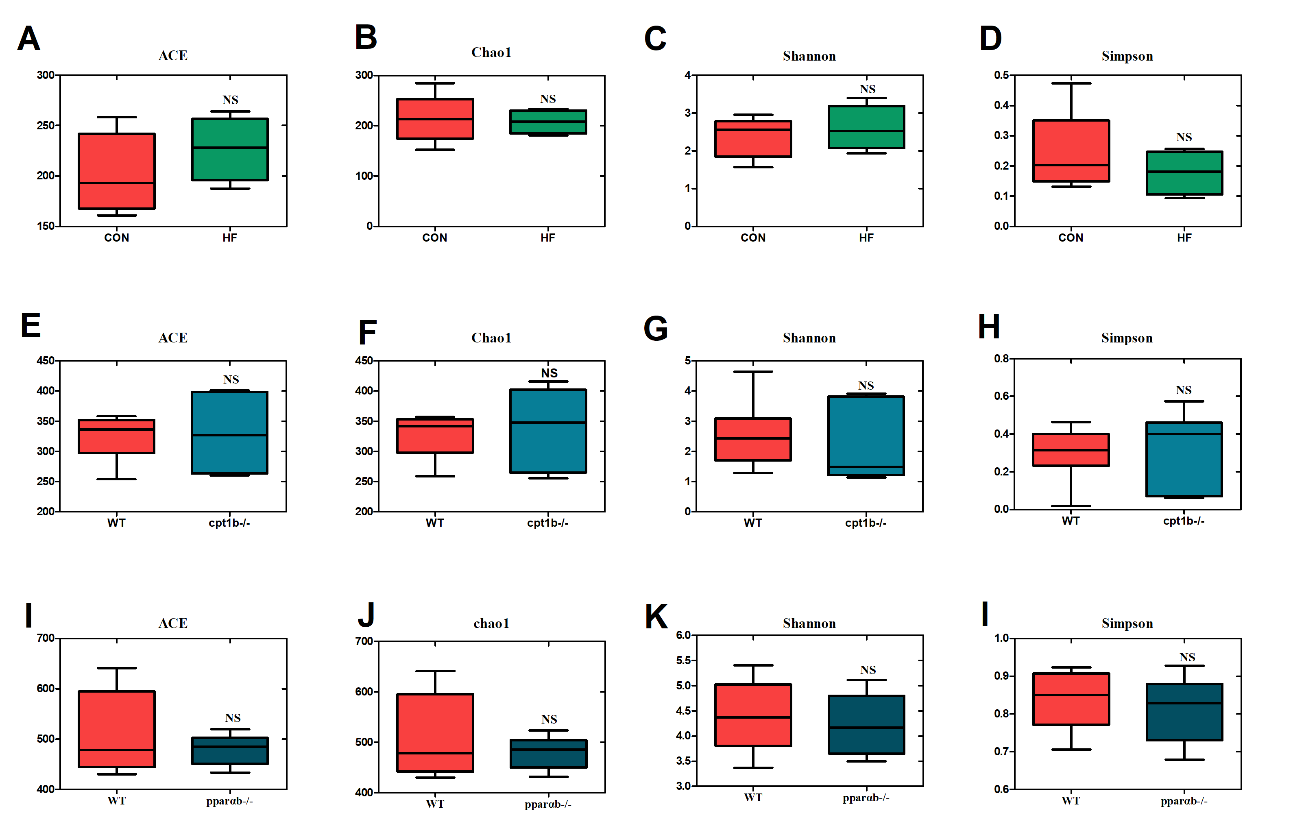
**

**Supplementary Figure 1**Boxplots of DNA-based α diversity indices of bacterial community based on DAN sequencing.**(A-D)**Gut microbial αdiversity of dietary groups based on the (**A**)ACE, (**B**)Chao1, (**C**)Shannon, (**D**)Simpson. **(E-H)**Gut microbial α diversity of WT and *cpt1b*^-/-^zebrafish groups based on the (**E**) ACE, (**F**) Chao1, (**G**)Shannon, (**H**)Simpson.**(I-L)**Gut microbial α diversity of WT and *pparab*^-/-^zebrafish based on the (**I**) ACE, (**J**) Chao1, (**K**)Shannon, (**L**)Simpson.
